# Isoxazole Derivative Induces Apoptosis-like Death and Autophagy through Oxidative Stress in *Leishmania amazonensis*

**DOI:** 10.1021/acsomega.5c11341

**Published:** 2026-05-02

**Authors:** Amanda Beatriz Kawano Bakoshi, Rayanne Regina Beltrame Machado, Karlos Eduardo Pianoski, Samara Mendes De Souza Melo, Fernanda Andreia Rosa, Sueli De Oliveira Silva Lautenschlager, Tania Ueda-Nakamura, Celso Vataru Nakamura, Danielle Lazarin-Bidóia

**Affiliations:** † Laboratory of Technological Innovation in the Development of Drugs and Cosmetics, 42487 State University of Maringá, 87020-900 Maringá, PR, Brazil; ‡ Department of Chemistry, State University of Maringá, 87020-900 Maringá, PR, Brazil

## Abstract

Leishmaniasis is one of the most important neglected tropical diseases and a significant global health burden, affecting millions of people worldwide. The limitations of current treatments, including toxicity, high cost, and the emergence of drug resistance, underscore the urgent need for new antileishmanial agents. Isoxazoles are a versatile class of heterocyclic compounds with documented antiparasitic activity, making them attractive scaffolds for drug development. In this study, we synthesized and evaluated a new isoxazole derivative, 4-[((4-fluorophenyl)­amino)­methyl]-5-(4-nitrophenyl)-3-[(2*E*)-*N*′-(2-pyridinylmethylene)­hydrazinecarbonyl]­isoxazole (**4**), against *Leishmania amazonensis*. Compound **4** exhibited potent activity, with IC_50_ values of 12.7 μM for promastigote forms and 0.96 μM for intracellular amastigotes, the most clinically relevant form, while displaying low cytotoxicity against J774A.1 macrophages and L929 fibroblasts, indicating a favorable safety profile. We further explored its mechanism of action through detailed analyses in promastigote and amastigote forms. Compound **4** induced oxidative stress, evidenced by elevated reactive oxygen species and nitric oxide content, leading to lipid peroxidation, lipid droplet accumulation, mitochondrial depolarization, and altered ATP levels. These effects were accompanied by loss of cell membrane integrity, reduced cell size, and phosphatidylserine exposure. An increase in autophagic vacuoles and acidic compartments was also observed. Morphological and ultrastructural alterations corroborated the biochemical findings. *In silico* analysis provided an initial drug-likeness and physicochemical context to support early stage prioritization. Collectively, our results demonstrate that compound **4** induces apoptosis-like cell death and autophagy through oxidative stress and mitochondrial dysfunction, highlighting it as a promising lead for the development of antileishmanial agents.

## Graphical abstract

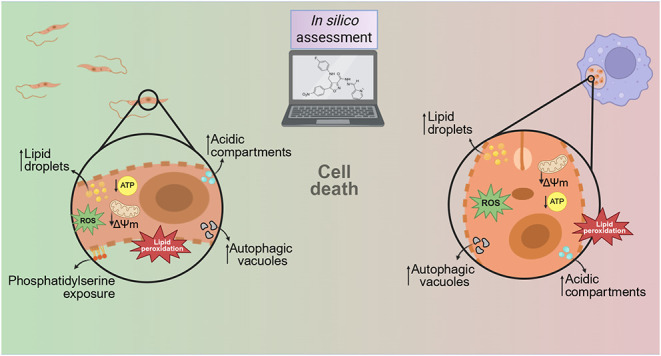

## 1 Introduction

Neglected tropical diseases (NTDs) comprise a diverse group of illnesses affecting over a billion people across 149 tropical and subtropical countries, significantly impairing the health and economic development of low-income nations.^1,2^ Despite their significant impact, NTDs frequently receive limited attention from public health authorities and researchers, resulting in inadequate funding, insufficient diagnostic methods, slow drug development, and lack of public health interventions.^3–5^ Among NTDs, leishmaniasis stands out, representing a group of infectious diseases endemic in 99 countries and territories caused by various species of obligate intracellular protozoa of the genus *Leishmania*.^6–8^ It is estimated that 700,000 to 1 million new cases occur each year,^9^ although underreporting remains a persistent challenge.^10,11^

The life cycle of *Leishmania* parasites involves alternating between vertebrate hosts and insect vectors.^12^ Clinical manifestations of leishmaniasis depend on complex interactions between the characteristics of the *Leishmania* species and the host immune response.^13,14^ *Leishmania amazonensis* is one of the causative agents of cutaneous leishmaniasis (CL), which can manifest in various clinical forms, including localized, disseminated, mucocutaneous, and diffuse.^15,16^ Although mortality from CL is generally low, the disease profoundly impacts economies and health systems, as well as the quality of life and mental health of patients, due to the social stigma associated with skin lesions and disfiguring scars.^17^

Current treatment of leishmaniasis relies primarily on pentavalent antimonials such as meglumine antimoniate and sodium stibogluconate. However, these drugs are associated with severe toxicity and require parenteral administration, which limits their use.^18^ Second-line drugs, including pentamidine, amphotericin B, paromomycin, and miltefosine, also present challenges related to toxicity and the emergence of drug resistance.^19^ Lipid formulations of amphotericin B have shown efficacy with reduced toxicity but remain costly, limiting accessibility.^20^ The rise of drug-resistant strains, combined with the limited number of therapeutic options, high toxicity of existing drugs, and insufficient investment in drug discovery, underscores the urgent need for new strategies to control and treat leishmaniasis.^18^

In this context, isoxazoles have emerged as attractive scaffolds in medicinal chemistry. These heterocyclic compounds, characterized by the presence of oxygen and nitrogen atoms in positions 1 and 2, are widely used in the pharmaceutical industry due to their broad spectrum of activities, including antibacterial, antiviral, antifungal, anti-inflammatory, and antitumor properties.^21^ Successful applications of isoxazole derivatives have led to several marketed drugs, underscoring the medicinal relevance of this heterocyclic scaffold. Examples include leflunomide (antirheumatic), sulfisoxazole (antibacterial), valdecoxib (anti-inflammatory), and risperidone (antipsychotic).^21,22^ Importantly, isoxazole derivatives have also demonstrated antileishmanial activity.^23–25^ In our previous study, we reported the synthesis of 3,4,5-trisubstituted isoxazoles that exhibited potent activity against *L. amazonensis* promastigotes.^25^ Within that framework, *para*-chloro substitution on the benzylamine ring was identified as a conservative handle to modulate activity, while highlighting the need to improve selectivity (Figure 1a).

**Figure 1:**
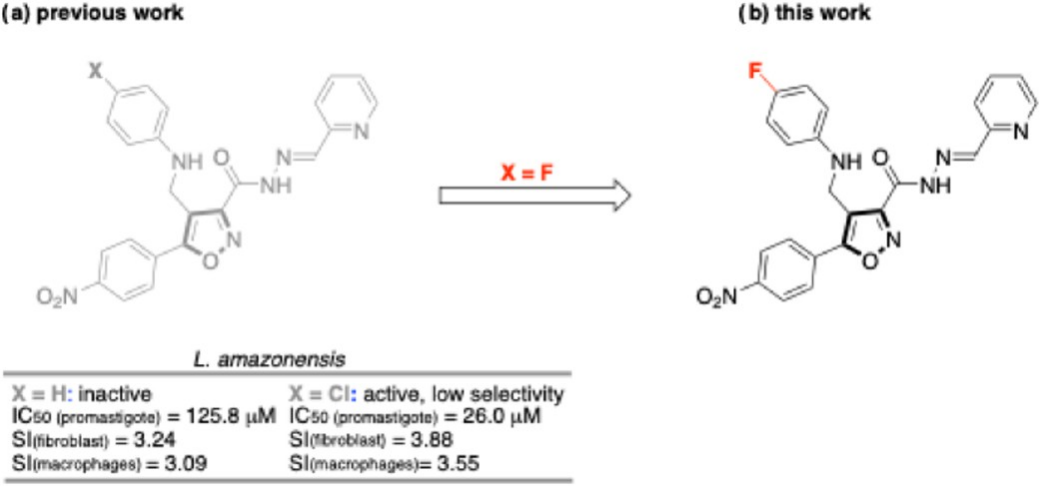
(a) The improvement of antileishmanial activity by the introduction of a chlorine atom in the isoxazole framework. (b) The analysis of the fluorinated isoxazole analogue (this work).

To address the need for novel antileishmanial agents, the present study describes the synthesis and biological evaluation a new 3,4,5-trisubstituted isoxazole, 4-[((4-fluorophenyl)­amino)­methyl]-5-(4-nitrophenyl)-3-[(2*E*)-*N*′-(2-pyridinylmethylene)­hydrazinecarbonyl]­isoxazole (**4**). Compound **4** was designed as a second-generation analogue of our previously reported 3,4,5-trisubstituted isoxazole framework using a hypothesis-driven, incremental modification strategy. Specifically, the *para*-fluoro substituent was introduced as a widely used lead-optimization modification to fine-tune aryl electronics and physicochemical properties with minimal steric impact. In many chemical series, *para*-fluorination provides an incremental modulation of lipophilicity and ionization-dependent permeability and may attenuate oxidative aromatic metabolism (“metabolic blocking”), thereby potentially improving cellular exposure without major conformational perturbation.^26^ In addition, a *para*-nitroaryl unit was maintained as a strong electron-withdrawing aromatic substituent frequently associated with oxidative-stress phenotypes in kinetoplastids.^27^ Finally, the 2-pyridyl *N*-acylhydrazone moiety was retained as a hypothesis-testing element to support subsequent mechanistic interrogation (*e*.*g*., mitochondrial/redox involvement), while recognizing that functional-group stability and liability should be experimentally addressed during further optimization (Figure 1b).

In this work, compound **4** was evaluated *in vitro* against promastigote and intracellular amastigote forms of *L. amazonensis*. Morphological, ultrastructural, and biochemical analyses were performed to characterize parasite responses and to support a working mechanistic interpretation of parasite death. These studies aim to provide phenotypic evidence for the antileishmanial potential of compound **4**.

## 2 Results

### 2.1 Synthesis of Isoxazole 4

The isoxazole **4** was synthesized following a simple and efficient four-step methodology starting from β-enamino diketones **1** and hydroxylamine (Scheme 1), as previously reported by us.^25^

**Scheme 1:**
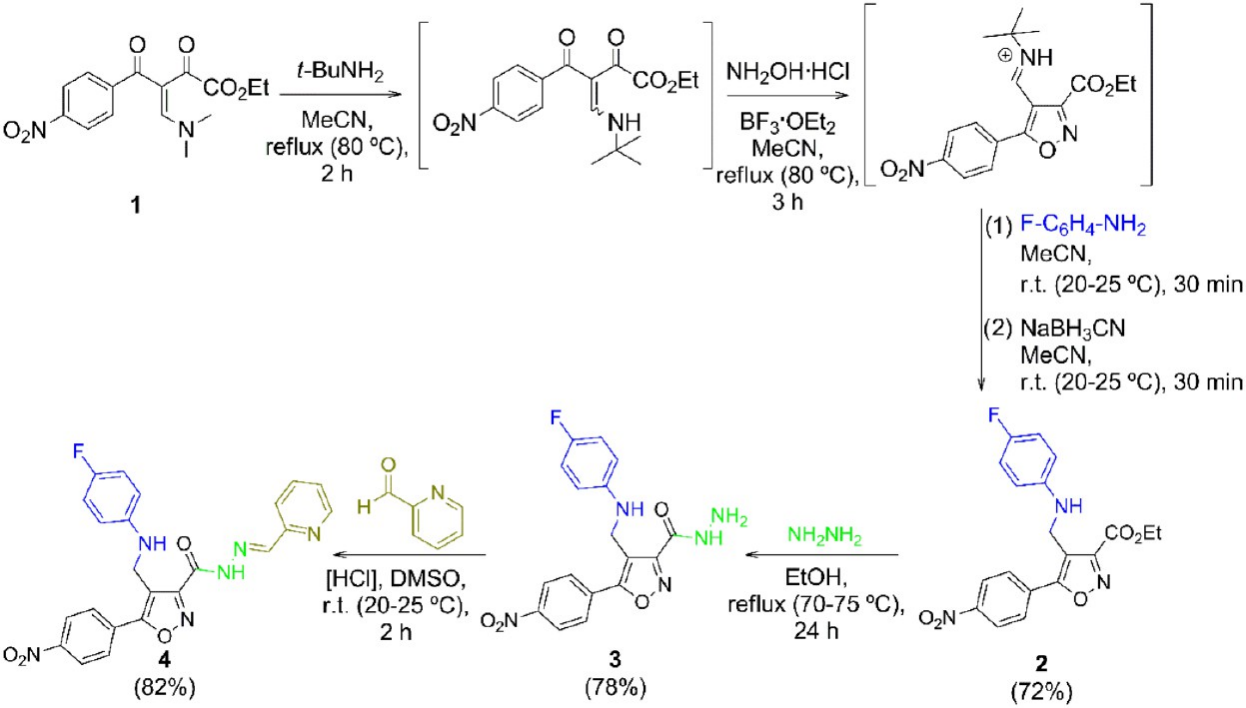
Synthesis of Isoxazole **4^a^,^b^**

a Reaction conditions: β-enamino diketone **1** (1.0 mmol), *t*-Bu-amine (1.05 mmol), MeCN (4.0 mL), hydroxylamine hydrochloride (1.2 mmol), BF_3_OEt_2_ (2.0 mmol), 4-fluoroaniline (3.0 mmol), NaBH_3_CN (1.2 mmol). Isoxazole **2** (1.0 mmol, 1.0 equiv), hydrazine monohydrate (20.0 mmol, 20.0 equiv), EtOH (4.0 mL). Isoxazole **3** (1.0 mmol), 2-formylpyridine (1.0 mmol), DMSO (2.0 mL), two drops of hydrochloric acid (37%).
b Isolated yield of isoxazole **2** after recrystallization in ethanol. Isolated yield of isoxazole **3** and **4** after filtration and washed with cold water.

### 2.2 Isoxazole **4** Selectively Inhibits the Proliferation of *L. amazonensis*

The investigation into the biological activity of isoxazole **4** against *L. amazonensis* revealed its selective inhibition of parasite proliferation. Isoxazole **4** inhibited proliferation of promastigotes, with an IC_50_ of 12.7 μM. Notably, compound **4** also demonstrated potent activity against the intracellular form of the parasite, exhibiting an IC_50_ of 0.96 μM. As illustrated in Figure 2, treatment resulted in a marked reduction of intracellular amastigotes (indicated by arrows) within the macrophages. To assess the safety profile, the cytotoxic effects of the compound were evaluated, revealing CC_50_ values of 196.1 μM against J774A.1 macrophages and 232.1 μM against L929 fibroblasts. These findings underscore the selectivity of compound **4** against intracellular amastigotes compared to mammalian cells, as evidenced by selectivity indexes of 204.27 for macrophages and 241.77 for fibroblasts. For comparison, miltefosine, a clinically approved antileishmanial drug, exhibits IC_50_ values of 20.75 μM against promastigotes and 1.82 μM against intracellular amastigotes of *L. amazonensis*, with a CC_50_ of 55.12 μM in J774A.1 macrophages, corresponding to a selectivity index of 30.2, as previously reported.^28^

**Figure 2:**
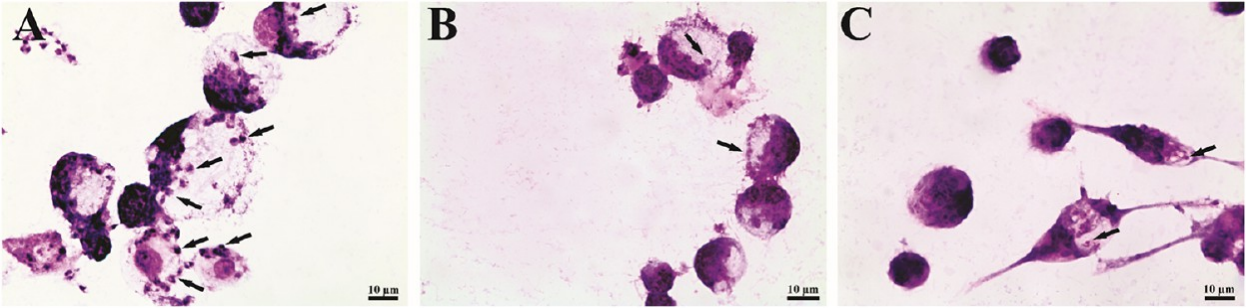
Optical microscopy of J774A.1 macrophages infected with *L. amazonensis* amastigotes (arrows) and treated with isoxazole **4** for 48 h. Untreated cells (A). Cells treated with 1 μM (B) and 0.1 μM (C) of isoxazole **4**. Scale bars: 10 μm.

### 2.3 Isoxazole 4 Causes Morphological and Ultrastructural Changes in *L. amazonensis*

Following the assessment of the activity of compound **4** against *L. amazonensis*, we investigated the morphological and ultrastructural changes caused by its treatment. Scanning electron microscopy (SEM) images revealed untreated promastigotes with typical cellular characteristics, such as elongated body and terminal flagellum (Figure 3A,B). These morphological features were altered in treated promastigotes, which displayed a reduction in cell body size, adopting a rounded morphology, accompanied by the loss of cellular content in the flagellar region and noticeable alterations in the cell surface (Figure 3C–F). Transmission electron microscopy (TEM) images showed untreated promastigotes with normal ultrastructure, characterized by intact organelles and nuclei (Figure 3A′,B′). In contrast, treatment with isoxazole **4** resulted in significant ultrastructural alterations in promastigotes, including mitochondrial swelling, accumulation of lipid bodies, and disruptions in nuclear integrity, such as disorganization and rupture of the nuclear membrane (Figure 3C′–F′). Regarding intracellular forms, SEM images depicted amastigotes with typical morphology (Figure 4A,B), while treatment with compound **4** led to a reduction in the number of intracellular amastigotes (Figure 4C–F). TEM analysis of treated amastigotes within macrophage parasitophorous vacuoles revealed notable alterations, including an increase in autophagic vacuoles and accumulation of lipid bodies (Figure 4C′–F′), compared to untreated amastigotes with normal cellular structures (Figure 4A′,B′).

**Figure 3:**
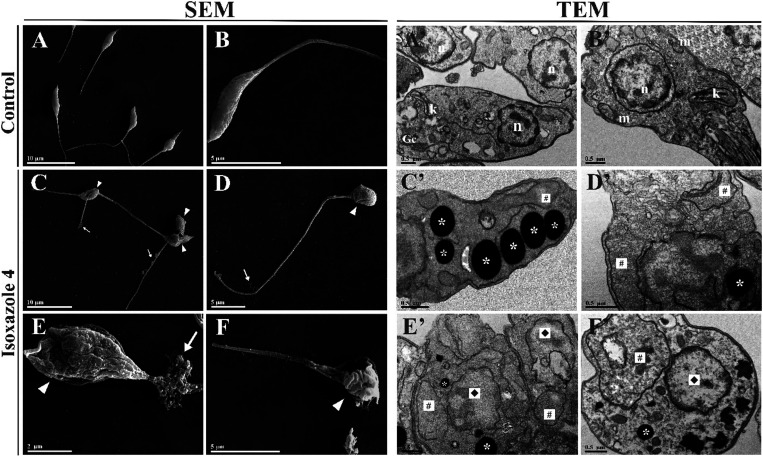
Morphological and ultrastructural alterations in *L. amazonensis* promastigotes treated with IC_50_ (12.7 μM) and 2 × IC_50_ (25.4 μM) of isoxazole **4** for 72 h, analyzed by scanning electron microscopy (SEM) (A–F) and transmission electron microscopy (TEM) (A′–F′). SEM images show untreated promastigotes with normal morphology (A, B). Promastigotes treated with IC_50_ of isoxazole **4** exhibit cell body reduction, rounding, cellular content leakage in the flagellar region (white arrows), and surface alterations (white arrowheads) (C–E), while those treated with 2 × IC_50_ display further cell size reduction, rounding, loss of cellular content, and surface alterations (white arrowheads) (F). TEM images reveal typical cellular structures in untreated promastigotes, including the kinetoplast (k), nucleus (n), Golgi complex (Gc) and mitochondrion (m) (A′, B′). Promastigotes treated with IC_50_ (C′–E′) and 2 × IC_50_ (F′) of isoxazole **4** show accumulation of lipid droplets (*), mitochondrial swelling (#), and nuclear alterations (⧫). Scale bars: 10 μm (A, C), 5 μm (B, D, F), 2 μm (E), 0.5 μm (A′–F′).

**Figure 4:**
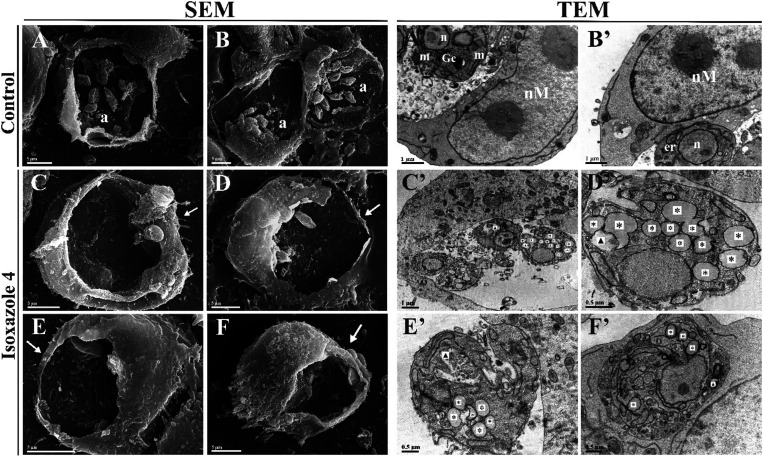
Morphological and ultrastructural alterations in intracellular amastigotes of *L. amazonensis* treated with IC_50_ (0.96 μM) and 2 × IC_50_ (1.92 μM) of isoxazole **4** for 48 h, analyzed by scanning electron microscopy (SEM) (A–F) and transmission electron microscopy (TEM) (A′–F′). SEM images show untreated amastigotes within the parasitophorous vacuole of macrophages with normal morphology (a) (A, B). A reduced number of amastigotes is observed following treatment with IC_50_ (C-E) and 2 × IC_50_ (F) of isoxazole **4** (white arrows). TEM images show intracellular amastigotes inside macrophages, with untreated parasites displaying typical cellular structures, including the endoplasmic reticulum (er), Golgi complex (Gc), nucleus (n), mitochondrion (m), and the macrophage nucleus (nM) (A′, B′). Treated amastigotes show accumulation of lipid droplets (*) and an increase in autophagic vacuoles (▶) at IC_50_ (C′–E′) and 2 × IC_50_ (F′). Scale bars: 5 μm (A–F), 1 μm (A′–C′), 0.5 μm (D′–F′).

### 2.4 Isoxazole **4** Causes Oxidative Stress in *L. amazonensis*

The assessment of ROS content was conducted employing fluorescence microscopy and fluorimetry techniques, utilizing the nonfluorescent marker H_2_DCFDA. Exposure to compound **4** resulted in a significant increase of total ROS levels in both promastigotes (Figure 5A) and amastigotes (Figure 5A′), compared to the control group. In promastigotes, this increase reached 168 and 319% when treated with the IC_50_ and 2 × IC_50_ concentrations of compound **4**, respectively (Figure 5B). Similarly, in amastigotes, the increase amounted to 402 and 475% with the IC_50_ and 2 × IC_50_ of compound **4**, respectively (Figure 5B′). Hydrogen peroxide (H_2_O_2_, 50 μM) also caused an increase in total ROS levels by 220% and 180% in promastigotes and amastigotes, respectively.

**Figure 5:**
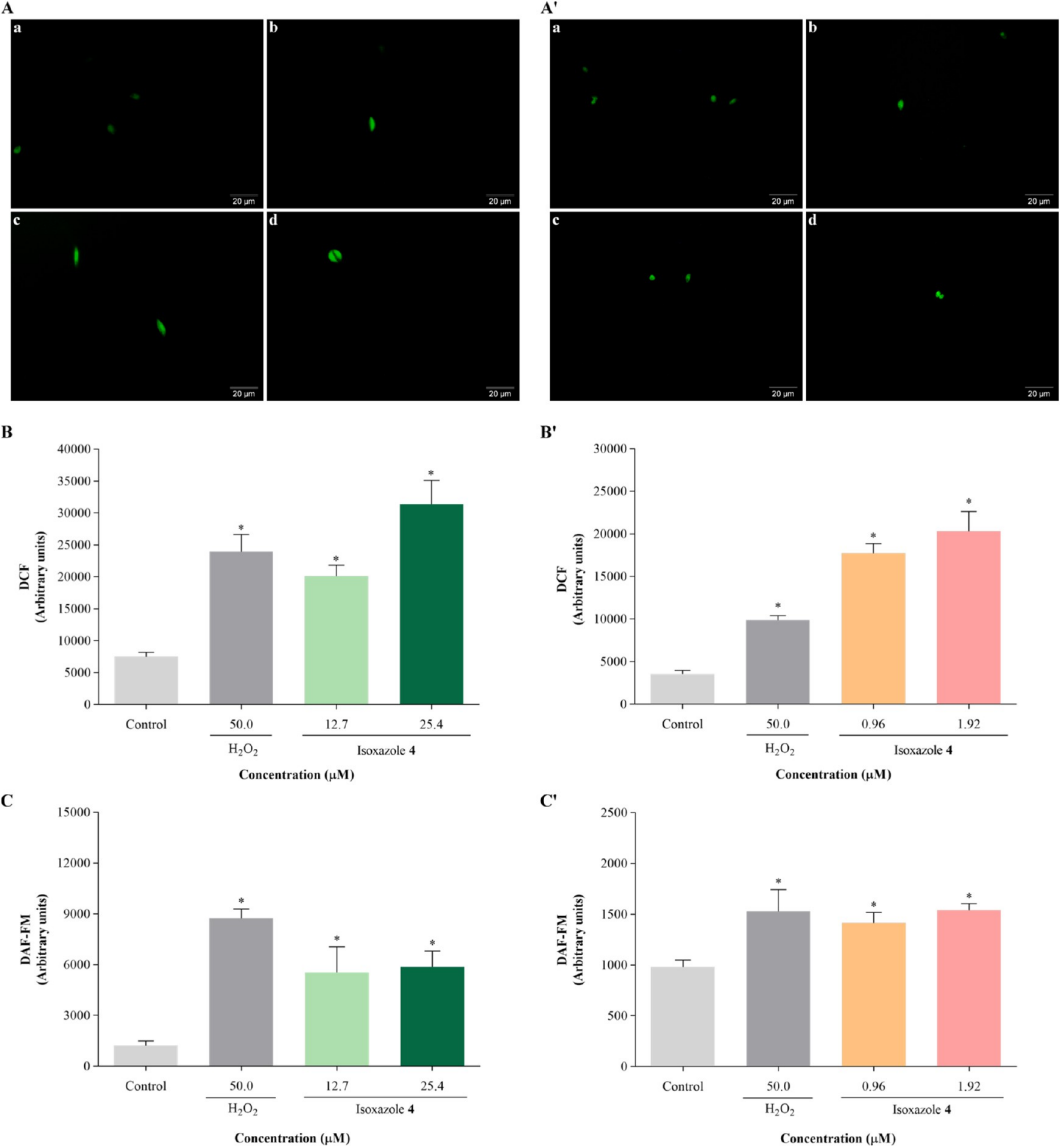
Isoxazole **4** induces oxidative stress in *L. amazonensis*. Parasites were treated with IC_50_ and 2 × IC_50_ of isoxazole **4** for 24 h. Reactive oxygen species content was evaluated using the nonfluorescent probe H_2_DCFDA: (A, B) promastigotes; (A′, B′) amastigotes. (A, A′) Fluorescence microscopy images: (a) untreated parasites, (b) parasites treated with 50 μM H_2_O_2_, (c) parasites treated with IC_50_ of isoxazole **4**, (d) parasites treated with 2 × IC_50_ of isoxazole **4**. Scale bars: 20 μm. (B, B′) Fluorescence intensity measured by fluorimetry. Nitric oxide content was evaluated using the DAF-FM DA probe: (C) promastigotes; (C′) amastigotes. H_2_O_2_ (50 μM) was used as positive control. Data are mean ± s.d. *P < 0.05 indicates significant differences compared to the control group. Statistical significance was determined by one-way ANOVA with Tukey’s multiple comparisons test.

Observing the rise in total ROS post-**4** treatment in parasites, the nitric oxide (NO) content was assessed using the DAF-FM DA marker to explore the potential induction of reactive nitrogen species (RNS) by the compound. Treatment with compound **4** promoted a significant increase in NO levels in promastigotes, exhibiting a 4.5-fold increase with the IC_50_ and a 4.8-fold increase with the 2 × IC_50_ of isoxazole **4**, in comparison to the control group (Figure 5C). A corresponding elevation in NO levels was observed in treated amastigotes, showing a 1.4-fold and 1.5-fold increase with the IC_50_ and 2 × IC_50_ concentrations of **4**, respectively (Figure 5C′). Similarly, H_2_O_2_ triggered an increase in NO levels by 7.1-fold and 1.5-fold in promastigotes and amastigotes, respectively.

### 2.5 Isoxazole **4** Induces Lipid Peroxidation and Lipid Droplets Accumulation in *L. amazonensis*

Lipid peroxidation in parasites subjected to compound **4** treatment was assessed using the nonfluorescent marker DPPP. Promastigotes treated with **4** exhibited a significant elevation in lipid peroxidation by 409 and 354% when treated with IC_50_ and 2 × IC_50_, respectively (Figure 6A). Similarly, in amastigotes, the increase amounted to 209 and 256% with IC_50_ and 2 × IC_50_, respectively, compared to the negative control (Figure 6A′). Exposure to H_2_O_2_ resulted in an increase in lipid peroxidation of 327 and 230% in promastigotes and amastigotes, respectively.

**Figure 6:**
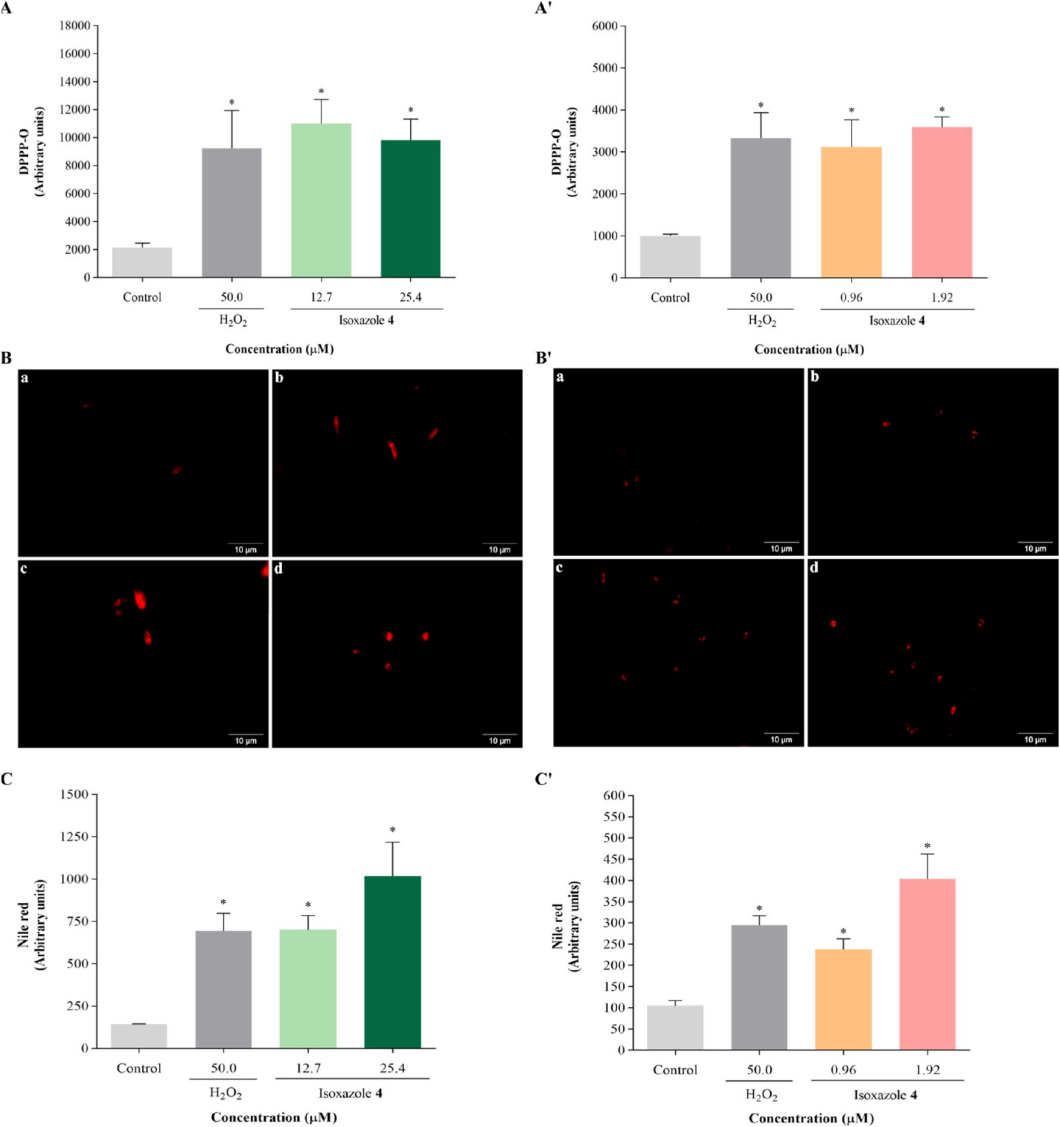
Isoxazole **4** induces lipid peroxidation and increases lipid droplets in *L. amazonensis*. Parasites were treated with IC_50_ and 2 × IC_50_ of isoxazole **4** for 24 h. Lipid peroxidation was assessed using the nonfluorescent marker DPPP: (A) promastigotes; (A′) amastigotes. Lipid droplets accumulation was assessed using Nile red staining: (B, C) promastigotes; (B′, C′) amastigotes. (B, B′) Fluorescence microscopy images: (a) untreated parasites, (b) parasites treated with 50 μM H_2_O_2_, (c) parasites treated with IC_50_ of isoxazole **4**, (d) parasites treated with 2 × IC_50_ of isoxazole **4**. Scale bars: 10 μm. (C, C′) Fluorescence intensity measured by fluorimetry. H_2_O_2_ (50 μM) was used as positive control. Data are mean ± s.d. *P < 0.05 indicates significant differences compared to the control group. Statistical significance was determined by one-way ANOVA with Tukey’s multiple comparisons test.

Accumulation of lipid droplets was assessed using the Nile red dye. Treatment with compound **4** resulted in significant increases in lipid bodies in promastigotes (Figure 6B) and amastigotes (Figure 6B′) compared to untreated cells. Promastigotes treated with IC_50_ and 2 × IC_50_ of **4** exhibited 4.9-fold and 7.0-fold increases in lipid bodies, respectively (Figure 6C). Similarly, in amastigotes, increases of 2.2-fold and 3.8-fold were observed with IC_50_ and 2 × IC_50_ of **4**, respectively (Figure 6C’). Furthermore, H_2_O_2_ also promoted lipid bodies accumulation, showing a 4.8-fold increase in promastigotes and 2.8-fold increase in amastigotes.

### 2.6 Isoxazole **4** Disrupts Mitochondrial Membrane Potential and Alters ATP Levels in *L. amazonensis*

Assessment of mitochondrial membrane potential (ΔΨm) was conducted by flow cytometry utilizing the Rh123 marker. Treatment-induced reductions in total fluorescence intensity of Rh123 were observed in promastigotes (Figure 7A) and amastigotes (Figure 7A′), indicating mitochondrial depolarization. Promastigotes treated with IC_50_ and 2 × IC_50_ of compound **4** exhibited reductions in ΔΨm by 30% and 41%, respectively (Figure 7B), while in amastigotes, reductions were recorded at 71 and 70%, respectively (Figure 7B′). Additionally, CCCP induced reductions in ΔΨm by 52% in promastigotes and 63% in amastigotes.

**Figure 7:**
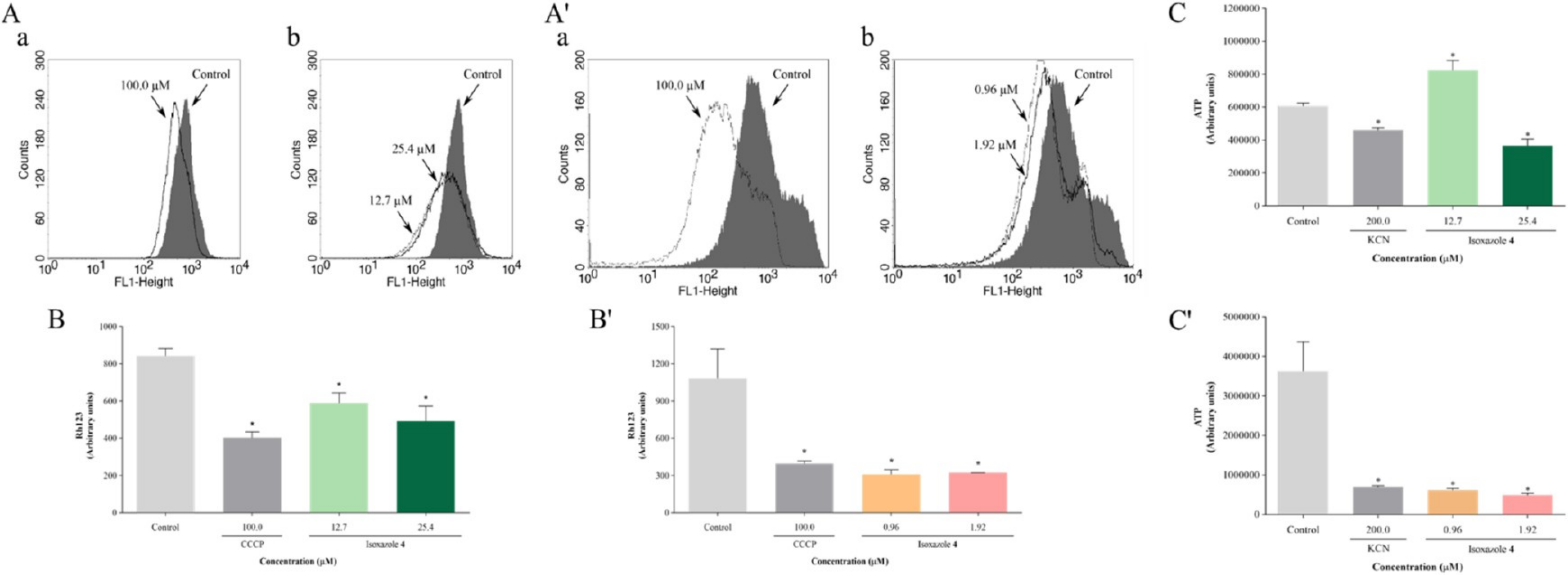
Isoxazole **4** causes mitochondrial dysfunction in *L. amazonensis*. Parasites were treated with IC_50_ and 2 × IC_50_ of isoxazole **4** for 24 h. Mitochondrial membrane potential was assessed using Rh123 staining: (A, B) promastigotes; (A′, B′) amastigotes. (A, A′) Histograms: (a) Parasites treated with 100 μM CCCP, (b) Parasites treated with IC_50_ and 2 × IC_50_ of isoxazole **4**. (B, B′) Fluorescence intensity. Intracellular ATP levels were assessed using the CellTiter-Glo reagent: (C) promastigotes; (C′) amastigotes. CCCP (100 μM) and KCN (200 μM) were used as positive controls for mitochondrial depolarization and ATP depletion, respectively. Data are mean ± s.d. *P < 0.05 indicates significant differences compared to the control group. Statistical significance was determined by one-way ANOVA with Tukey’s multiple comparisons test.

Intracellular ATP levels were quantified using the CellTiter-Glo reagent. Promastigotes treated with IC_50_ of **4** displayed a 35% increase in ATP levels, contrasting with a 40% decrease observed with 2 × IC_50_ treatment (Figure 7C). In amastigotes, treatment with compound **4** caused a decrease in ATP levels of 83 and 86% with IC_50_ and 2 × IC_50_, respectively, compared to the negative control (Figure 7C′). KCN, an inhibitor of the cytochrome oxidase complex of the electron transport chain, induced decreases in ATP levels by 24 and 80% in promastigotes and amastigotes, respectively.

### 2.7 Isoxazole **4** Promotes Cell Size Reduction in *L. amazonensis* and Phosphatidylserine Externalization in Promastigotes

Cell size was evaluated by flow cytometry. Results revealed a significant decrease in cell size, by 57 and 66%, in promastigotes treated with IC_50_ and 2 × IC_50_ of compound **4**, respectively, compared to the control group (Figure 8A). Similar reductions were observed in amastigotes, with decreases in cell size by 50 and 49% when treated with IC_50_ and 2 × IC_50_ of **4**, respectively (Figure 8A′). Miltefosine treatment also resulted in decreased cell size, by 69 and 74% in promastigotes and amastigotes, respectively.

**Figure 8:**
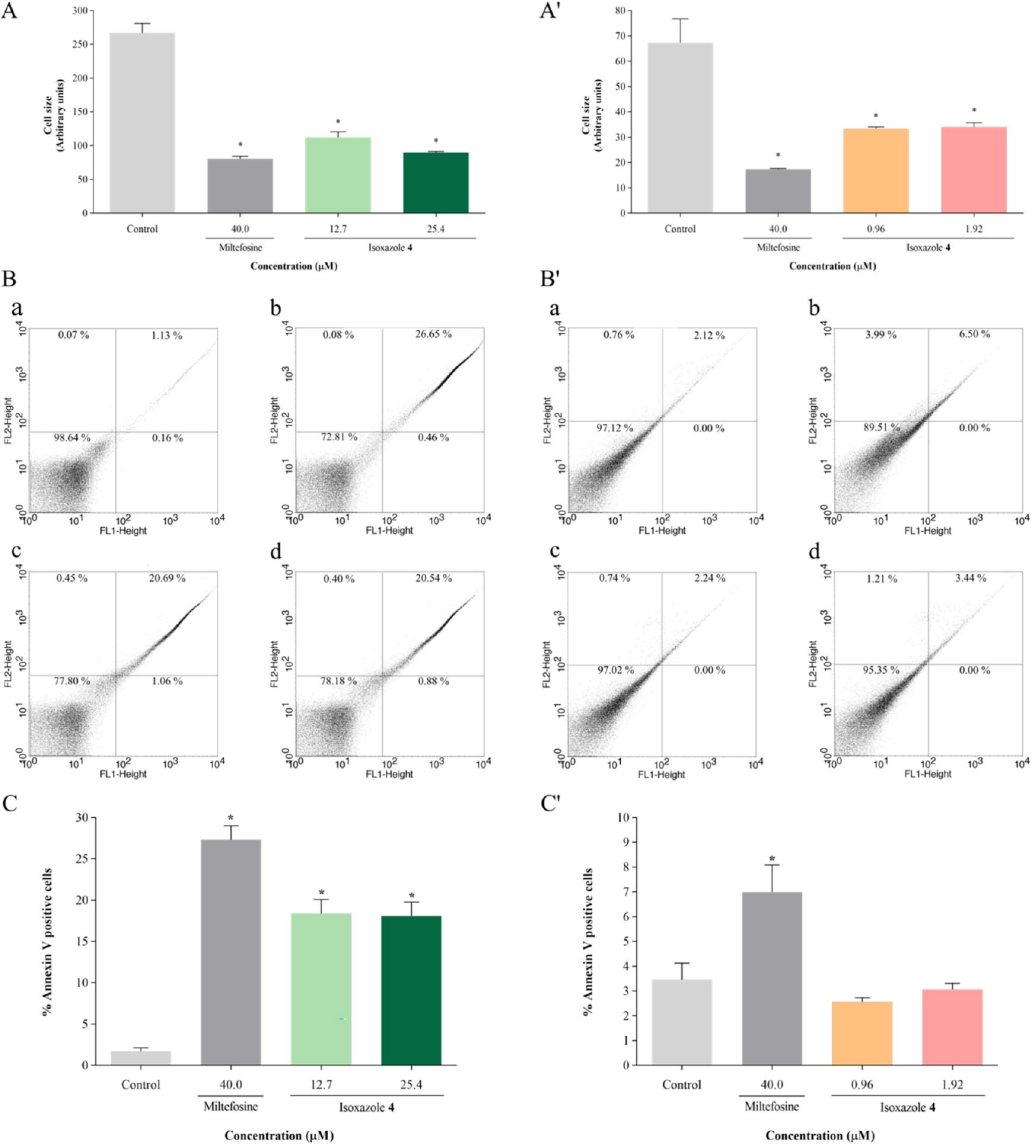
Isoxazole **4** induces cell size decrease in *L. amazonensis* and phosphatidylserine externalization in promastigotes. Parasites were treated with IC_50_ and 2 × IC_50_ of isoxazole **4** for 24 h. FSC-H was used as an indicator of cell size: (A) promastigotes; (A′) amastigotes. Phosphatidylserine exposure was assessed using Annexin V-FITC and PI staining: (B, C) promastigotes; (B′, C′) amastigotes. (B, B′) Dot plots: (a) untreated parasites, (b) parasites treated with 40 μM miltefosine, (c) parasites treated with IC_50_ of isoxazole **4**, (d) parasites treated with 2 × IC_50_ of isoxazole **4**. (C, C′) Percentage of Annexin V positive cells. Miltefosine (40 μM) was used as positive control. Data are mean ± s.d. *P < 0.05 indicates significant differences compared to the control group. Statistical significance was determined by one-way ANOVA with Tukey’s multiple comparisons test.

Assessment of phosphatidylserine externalization, a hallmark of apoptosis,^29^ was performed using Annexin V-FITC staining. Promastigotes treated with IC_50_ and 2 × IC_50_ of **4** exhibited notable increases in the percentage of Annexin V positive cells, indicative of phosphatidylserine externalization, by 11.0 and 10.8 times, respectively (Figure 8B,C), suggesting that isoxazole **4** induced an apoptosis-like cell death in promastigotes. Conversely, there were no increases in amastigotes (Figure 8B′,C′). Treatment with miltefosine resulted in increases of 16.4 and 2.0 times in the percentage of Annexin V positive cells in promastigotes and amastigotes, respectively.

### 2.8 Isoxazole **4** Induces Loss of Cell Membrane Integrity in *L. amazonensis*

Cell membrane integrity was assessed using PI staining. Promastigotes and amastigotes treated with **4** exhibited increased percentages of PI-positive cells, indicating cell membrane rupture. Promastigotes treated with IC_50_ and 2 × IC_50_ of compound **4** displayed respective PI fluorescence intensity increases of 13.3 and 39.9 times (Figure 9A,B), while in amastigotes, these increases were 3.4 and 3.9 times, respectively (Figure 9A′,B′). Digitonin also induced significant increases in fluorescence intensity of 62.9 times and 11.9 times in both promastigotes and amastigotes, respectively.

**Figure 9:**
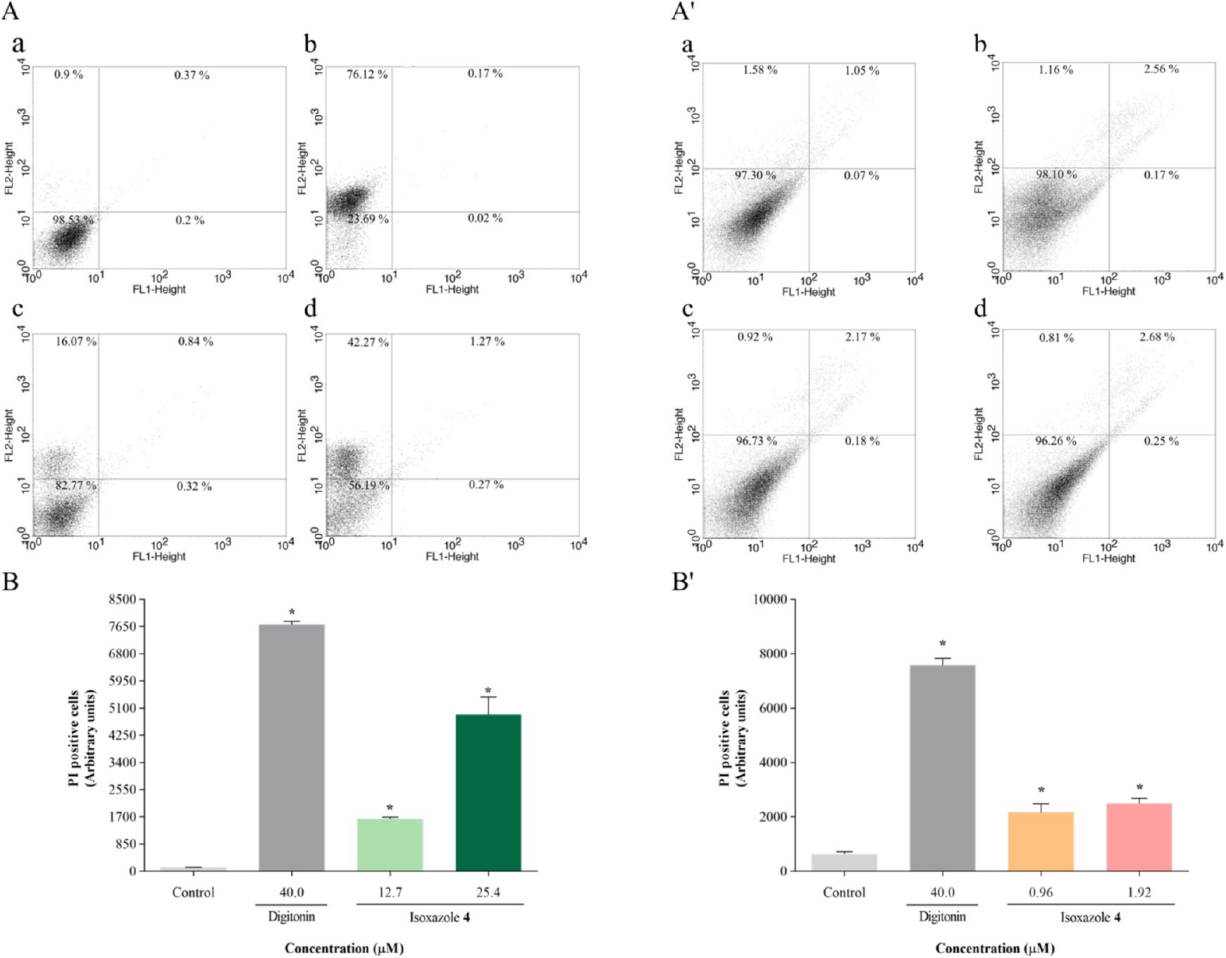
Isoxazole **4** induces plasma membrane permeabilization in *L. amazonensis*. Parasites were treated with IC_50_ and 2 × IC_50_ of isoxazole **4** for 24 h. Cell membrane integrity was assessed using PI staining: (A, B) promastigotes; (A′, B′) amastigotes. (A, A’) Dot plots: (a) untreated parasites, (b) parasites treated with 40 μM digitonin, (c) parasites treated with IC_50_ of isoxazole **4**, (d) parasites treated with 2 × IC_50_ of isoxazole **4**. (B, B′) PI-positive cells. Digitonin (40 μM) was used as positive control. Data are mean ± s.d. *P < 0.05 indicates significant differences compared to the control group. Statistical significance was determined by one-way ANOVA with Tukey’s multiple comparisons test.

### 2.9 Isoxazole **4** Increases Autophagic Vacuoles Formation and Acidic Compartments in *L. amazonensis*

Assessment of autophagic vacuoles was performed using fluorescence microscopy and fluorimetry with the fluorescent marker MDC. Treatment with compound **4** led to significant increases in autophagic vacuole formation in both promastigotes (Figure 10A) and amastigotes (Figure 10A′), compared to the control group. Promastigotes treated with IC_50_ and 2 × IC_50_ of **4** exhibited respective increases in autophagic vacuoles by 4.2 and 10.6 times. Parasites subjected to nutritional scarcity, induced by diluting the culture medium with PBS (positive control), showed a notable 4.5-fold increase in the formation of autophagic vacuoles. This effect was attenuated by the autophagy inhibitor, wortmannin, which inhibited autophagic vacuole formation by 66, 50, and 80% in promastigotes treated with positive control, IC_50_, and 2 × IC_50_, respectively (Figure 10B). In amastigotes, treatment with isoxazole **4** caused an increase in autophagic vacuoles of 2.0 times and 2.2 times with IC_50_ and 2 × IC_50_; similarly, the positive control led to a 1.8-fold increase. Wortmannin prevented the formation of autophagic vacuoles by 18, 34, and 49% in amastigotes treated with positive control, IC_50_, and 2 × IC_50_ (Figure 10B′).

**Figure 10:**
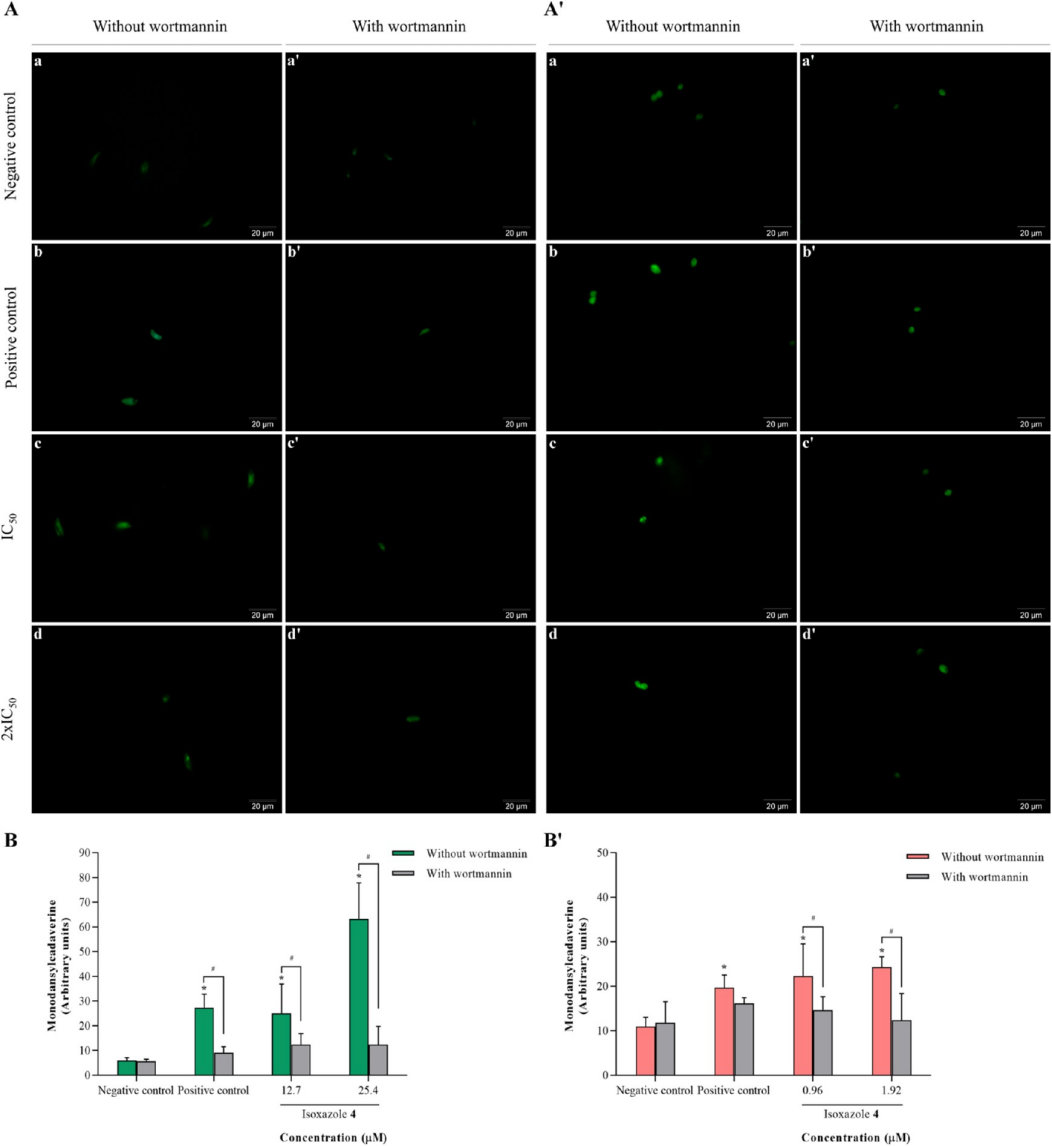
Isoxazole **4** increases the amount of autophagic vacuoles in *L. amazonensis*. Parasites were treated with IC_50_ and 2 × IC_50_ of isoxazole **4** for 24 h. Autophagic vacuoles accumulation was evaluated using MDC staining: (A, B) promastigotes; (A′, B′) amastigotes. (A, A′) Fluorescence microscopy images: (a) untreated parasites, (a′) untreated parasites plus wortmannin, (b) positive control, (b′) positive control plus wortmannin, (c) parasites treated with IC_50_ of isoxazole **4**, (c’) parasites treated with IC_50_ of isoxazole **4** plus wortmannin, (d) parasites treated with 2 × IC_50_ of isoxazole **4**, (d′) parasites treated with 2 × IC_50_ of isoxazole **4** plus wortmannin. Scale bars: 20 μm. (B, B′) Fluorescence intensity measured by fluorimetry. Parasites incubated with PBS in the absence of serum were used as positive control. Data are mean ± s.d. *P < 0.05 indicates significant differences compared to the control group. #*P* < 0.05 indicates significant differences between groups with and without wortmannin. Statistical significance was determined by two-way ANOVA with Tukey’s multiple comparisons test.

The presence of acidic vacuoles was evaluated using the acridine orange marker. The scatter plots show that the cell population is concentrated in the lower right quadrant, as the marker intercalates with DNA, emitting green fluorescence. This profile is altered after treatment with compound **4**, with an increase in cells in the upper right quadrant, indicating an elevation in red fluorescence emission, indicative of an increase in acidic compartments in the cells. Promastigotes treated with IC_50_ and 2 × IC_50_ of **4** displayed respective increases of 740 and 1054% in the red/green fluorescence ratio (Figure 11A,B). In amastigotes, increases of 99 and 108% were observed with IC_50_ and 2 × IC_50_ of **4**, respectively (Figure 11A′,B′). The positive control induced increases of 371 and 104% in promastigotes and amastigotes, respectively.

**Figure 11:**
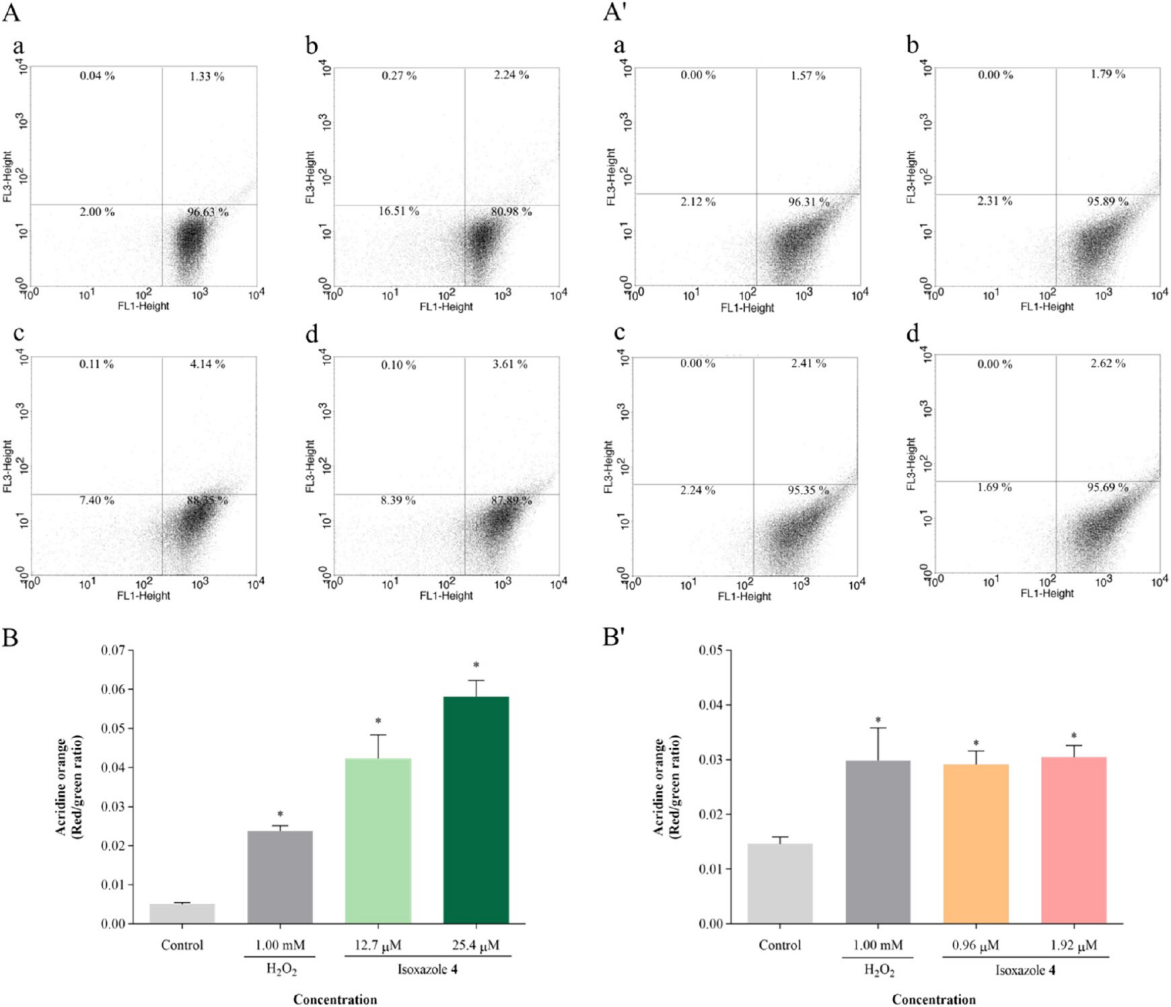
Isoxazole **4** induces an increase in acidic vacuoles in *L. amazonensis*. Parasites were treated with IC_50_ and 2 × IC_50_ of isoxazole **4** for 24 h. Acidic vacuoles formation was assessed using acridine orange staining: (A, B) promastigotes; (A′, B′) amastigotes. (A, A′) Dot plots: (a) untreated parasites, (b) parasites treated with 50 μM H_2_O_2_, (c) parasites treated with IC_50_ of isoxazole **4**, (d) parasites treated with 2 × IC_50_ of isoxazole **4**. (B, B′) Red/green fluorescence ratio in cells. H_2_O_2_ (50 μM) was used as positive control. Data are mean ± s.d. *P < 0.05 indicates significant differences compared to the control group. Statistical significance was determined by one-way ANOVA with Tukey’s multiple comparisons test.

### 2.10 *In Silico* Predictions of Drug-like Properties for Isoxazole **4**

Physicochemical descriptors and ADME-related predictions for compound **4** were generated using the SwissADME web tool^30^ (including the Bioavailability Radar, the BOILED-Egg model, P-gp substrate prediction, and medicinal-chemistry filters such as PAINS). These outputs should be interpreted as *in silico* estimates intended to support early stage hypothesis generation and prioritization, and not as experimental confirmation of developability or oral exposure. Lipinski’s and Veber’s criteria were analyzed to contextualize the compound within the commonly used “drug-like” chemical space (Table 1).^31,32^ While compound **4** meets these thresholds, compliance with empirical rules does not establish oral bioavailability. The SwissADME Bioavailability Score (0.55) and the Bioavailability Radar (Figure S3A) provide a heuristic overview across six physicochemical dimensions (lipophilicity, flexibility, polarity, size, solubility, and saturation). Pharmacokinetics-related predictions were further assessed using the BOILED-Egg model (Figure S3B), which suggested low gastrointestinal absorption and no passive BBB permeation for compound **4**.^33^ SwissADME also predicts that compound **4** is not a P-gp substrate (Table 1). Finally, the SwissADME PAINS filter returned no alerts for **4**; however, PAINS/structural-alert filters are heuristic screens and do not exclude all potential liabilities. In particular, the presence of an *N*-acylhydrazone functionality warrants careful interpretation and should be complemented by orthogonal biological assays and chemical stability assessment under relevant conditions.

**Table 1:** *In Silico* ADME and Physicochemical Profile of Isoxazole **4** (SwissADME)^a^

	isoxazole **4**	limits
molecular weight	460.42 g/mol	≤500
M Log P	1.42	≤4.15
hydrogen bond acceptors	8	≤10
hydrogen bond donors	2	≤5
TPSA	138.23 Å^2^	≤140 Å^2^
number of rotatable bonds	9	≤10
bioavailability score	0.55	-
blood-brain barrier permeant	No	-
gastrointestinal absorption	Low	-
P-gp substrate	No	-
water solubility	Moderate	-
PAINS	0	-

a All parameters were predicted using SwissADME. GI absorption and BBB permeation were estimated with the BOILED-Egg model; P-gp substrate and PAINS alerts correspond to the models/filters implemented in SwissADME.

## 3 Discussion

Numerous studies have been conducted to develop new drugs and strategies for treating patients with leishmaniasis.^34–36^ However, the high toxicity, severe side effects, administration challenges, and drug resistance observed with current treatments underscore the urgent need for more effective and safer compounds. Given the significant toxicity associated with leishmaniasis treatment, investigating the toxicity of compounds with antileishmanial activity in host cells is crucial. Our results demonstrate the activity of a novel isoxazole derivative (**4**) against *L. amazonensis* promastigotes, with low cytotoxicity against J774A.1 macrophages and L929 fibroblasts.

Macrophages serve as crucial host cells for *Leishmania* parasites, providing an environment where the parasites can proliferate and survive. Among the different morphological forms of the parasite, amastigotes are particularly significant, found mainly in macrophages.^37,38^ The effectiveness of chemotherapy for leishmaniasis has been limited by the location of intracellular amastigotes within the parasitophorous vacuole of macrophages. This vacuole acts as a protective shield against host defense mechanisms and antimicrobial compounds,^39^ highlighting the importance of compounds that can traverse host cell membranes and maintain stability within the acidic environment of the vacuole.

Given these considerations, we investigated the activity of compound **4** against intracellular amastigotes within J774A.1 macrophages. Our results demonstrated the high selectivity of **4** against intracellular amastigotes, exhibiting superior efficacy compared to promastigotes. This differential susceptibility aligns with previous findings.^40,41^ Importantly, the robust intracellular activity provides experimental support that compound **4** reaches and affects the intracellular parasite under our assay conditions.

To further contextualize the antileishmanial activity and selectivity of compound **4**, we benchmarked our findings against miltefosine, a well-established antileishmanial therapy. While both compounds display low micromolar activity against intracellular amastigotes of *L. amazonensis*, compound **4** presents a substantially higher selectivity index under the same *in vitro* conditions. This difference reflects a wider quantitative separation between antileishmanial activity and cytotoxicity for compound **4** relative to miltefosine. Taken together, these data indicate that compound **4** combines potent antileishmanial activity with a more favorable selectivity profile, supporting its potential as a lead compound for further investigation.

Subsequently, we investigated parasite responses and cell-death–associated phenotypes induced by compound **4**. Morphological and ultrastructural analyses revealed significant alterations, including decreased intracellular amastigotes in macrophages, reduced cell size, leakage of intracellular content, and surface alterations in promastigotes. Furthermore, treatment led to structural changes, such as increased lipid droplets, nuclear alterations, and mitochondrial swelling in promastigotes. The accumulation of lipid droplets was also a consequence of treatment with **4** in amastigotes, and the increase in autophagic vacuoles was also highlighted. Comparable phenotypic signatures have been reported for other antikinetoplastid compounds, pointing to oxidative stress–coupled mitochondrial dysfunction as a recurrently exploited vulnerability in *Leishmania* spp. Bortoleti et al.^42^ showed that acetyl-thiohydantoin derivatives trigger ROS overproduction alongside mitochondrial alterations, lipid body accumulation, and autophagic responses, closely mirroring the biochemical and ultrastructural changes observed here for compound **4**. Consistent with this phenotype-driven framework, Garcia et al.^43^ reported apoptosis-like death in *L. amazonensis* associated with mitochondrial dysfunction and ATP imbalance, reinforcing the relevance of mitochondrial stress pathways in parasite killing. Extending these observations to the clinically relevant intracellular stage, Paula et al.^44^ demonstrated that β-carboline derivatives eliminate intracellular amastigotes in macrophage infection models, with parasite death linked to oxidative stress and pronounced cellular alterations.

Given the pronounced phenotypic alterations observed, compound **4** was assessed for its ability to generate ROS and NO. Elevated ROS and NO levels were observed in treated promastigotes and amastigotes, suggesting induction of oxidative and nitrosative stress. Free radicals contain one or more unpaired electrons, enabling them to react with other molecules by donating or accepting their unpaired electron, thus increasing their stability. Consequently, ROS and RNS can be detrimental to various biological macromolecules, such as lipids, proteins, and nucleic acids, leading to mitochondrial dysfunction and ultimately cell death.^45–47^ Cell membranes and organelle membranes are particularly susceptible to damage induced by ROS due to their high content of polyunsaturated fatty acids. Lipid peroxidation caused by ROS leads to direct damage to phospholipids, resulting in alterations in membrane structure, affecting its fluidity, and compromising its integrity.^45,48^ Lipid peroxidation and loss of cell membrane integrity were observed in parasites treated with **4**. Furthermore, lipid peroxidation can also signal cell death, inducing programmed cell death.^48^

While hundreds of mitochondria may be found in mammalian cells, in trypanosomatids, this organelle is a unique tubular structure that extends throughout the cell body. In organisms with a single mitochondrion, their survival relies on its proper functioning, and the maintenance of the ΔΨm is crucial for cell viability.^49,50^ Mitochondrial dysfunction, indicated by mitochondrial depolarization and swelling, was evident in compound **4**-treated parasites, as verified by flow cytometry and TEM. ΔΨm disruption is characteristic of apoptosis in pathogenic trypanosomatids.^51^ Moreover, alterations in ATP levels in treated parasites further underscored mitochondrial dysfunction, highlighting the critical role of ΔΨm in cell survival.

The relationship between mitochondria and reactive species may be reciprocal, with mitochondrial dysfunction leading to ROS/RNS imbalance, but high ROS/RNS content can also lead to mitochondrial dysfunction.^52^ Notably, mitochondrial DNA is susceptible to oxidative damage, amplifying oxidative stress and apoptotic pathways.^53^

Accumulation of lipid bodies, observed in treated promastigotes and amastigotes, may reflect a cellular response to oxidative stress, serving as a protective mechanism against lipid peroxidation and facilitating long-term lipid supply for energy and cell survival.^54^ The rapid accumulation of lipid bodies in the cytoplasm is a characteristic feature of apoptosis.^55^ Additionally, other cellular changes indicative of apoptosis were observed, such as reduced cell size and phosphatidylserine externalization.^56^

The disruption of the plasma membrane was observed in parasites treated with **4**, as evidenced by PI staining, which is a characteristic feature of necrotic cell death. Oxidative stress in trypanosomatids may also be associated with necrosis: if elevated levels of ROS are produced, protozoa lysis and subsequent cell death by necrosis may occur.^51^ High levels of ROS and damage to the cell membrane have been highlighted in previous studies.^42,57^

Furthermore, the generation of ROS can also stimulate autophagy.^58^ Ultrastructural analysis is a crucial tool for characterizing autophagic phenotypes, and the observation of large vacuoles and damaged organelles in trypanosomatids may indicate that autophagy process is occurring in these cells.^51,59^ Indeed, trypanosomatids possess a functional autophagic system that appears to be essential for parasite differentiation and survival.^60^ However, in the case of extensive autophagic activity that destroys a large portion of the cytosol and organelles, there may be a total collapse of cellular functions.^61^ Compound **4** treatment caused an increase of autophagic vacuoles as demonstrated by fluorimetry and TEM. Wortmannin partially inhibited fluorescence, corroborating that the increase in MDC fluorescence was due to the formation of autophagic vacuoles. Autophagy induction further supports the involvement of oxidative stress in cellular responses to compound **4**. Prior studies have observed this effect.^28,42^

Acidic compartments, such as acidocalcisomes, are organelles involved in storing calcium, iron, sodium, magnesium, zinc, phosphorus compounds, and play a crucial role in maintaining intracellular pH homeostasis and osmoregulation in response to osmotic stress.^62^ Isoxazole **4** treatment caused an increase in acidic compartments, which suggests disruption of cellular homeostasis.

Importantly, the mechanistic features observed here are consistent with the original design rationale, in which the 2-pyridyl *N*-acylhydrazone moiety was intentionally retained as a hypothesis-testing element to probe redox- and mitochondria-associated stress responses. *N*-acylhydrazones can coordinate to metals under relevant conditions, and perturbing metal-dependent pathways represents a plausible route to disrupt mitochondrial function and redox homeostasis in kinetoplastids. In line with this hypothesis, related hydrazone/*N*-acylhydrazone scaffolds have been reported to promote mitochondrial depolarization and ROS accumulation in *Leishmania*,^63,64^ supporting phenotypic convergence with the response profile observed for compound **4**.

Beyond morphology and ROS measurements, the present study integrates complementary readouts spanning mitochondrial membrane potential, ATP imbalance, lipid peroxidation, phosphatidylserine exposure, plasma-membrane permeabilization, and autophagic vacuole formation. Collectively, these data support a coherent, hypothesis-generating framework in which oxidative stress and mitochondrial dysfunction are coupled stress responses associated with the apoptosis-like and autophagic phenotypes observed. At this stage, the mechanism of action of compound **4** should be interpreted as phenotypic rather than target-confirmed; no specific molecular target is identified in the present study, and definitive target assignment remains a goal for future investigations.

From a developability perspective, compound **4** fulfills Lipinski’s and Veber’s criteria and shows a SwissADME Bioavailability Score of 0.55; nevertheless, the BOILED-Egg model predicts low gastrointestinal absorption, and SwissADME indicates moderate water solubility. Therefore, these *in silico* results should be regarded as an initial physicochemical/ADME snapshot that may inform future optimization, rather than as evidence supporting any particular route of administration at this stage. P-gp is an ATP-dependent efflux transporter that can reduce absorption and intracellular exposure of its substrates.^65,66^ SwissADME predicts that compound **4** is not a P-gp substrate, which, if experimentally confirmed, could be favorable for cellular exposure. In addition, the predicted lack of passive BBB permeation suggests limited central nervous system (CNS) exposure, which may reduce CNS-related liabilities for a peripheral antiparasitic indication.^67^

Although SwissADME returned no PAINS alerts, PAINS/structural-alert filters are heuristic and should not be overinterpreted as definitive derisking. Importantly, the presence of an *N*-acylhydrazone motif has been discussed in the assay-interference/PAINS context in the medicinal chemistry literature and may also raise questions regarding chemical stability under acidic conditions.^68^ Accordingly, chemical stability in simulated gastric fluid (SGF) (and, optionally, in neutral/intestinal media) monitored by HPLC or LC–MS is a key next step before proposing an oral administration strategy.

In conclusion, we demonstrated the promising activity of the isoxazole **4** against *L. amazonensis*, coupled with its low cytotoxicity in J774A.1 macrophages and L929 fibroblasts. Notably, **4** exhibited high selectivity against intracellular amastigotes within macrophages, indicating its potential clinical relevance in targeting this crucial parasitic form. To gain deeper insights into the compound’s effects on the parasite, we investigated its mechanisms of action, revealing that isoxazole **4** induces morphological and ultrastructural alterations, and disrupts cellular homeostasis through oxidative stress and mitochondrial dysfunction. These findings highlight the compound’s broad impact on parasite physiology. Our results suggest that compound **4** induces apoptosis-like cell death and autophagy. Collectively, these findings position compound **4** as a promising phenotypic lead and provide a strong foundation for follow-up studies aimed at refining the working mechanistic hypothesis with orthogonal assays and derisking key developability questions, including chemical stability of the *N*-acylhydrazone motif under relevant conditions, prior to advancement into *in vivo* efficacy models.

## 4 Methods

### 4.1 Materials

Folic acid, 3-chlorophenylhydrazone carbonyl cyanide (CCCP), potassium cyanide (KCN), 2′,7′-dichlorodihydrofluorescein diacetate (H_2_DCFDA), digitonin, dimethyl sulfoxide (DMSO), hemin, miltefosine, monodansylcadaverine (MDC), rhodamine 123 (Rh123), wortmannin, 9-(diethylamino)-5*H*-benzo­[*a*]­phenoxazin-5-one (Nile red) and acridine orange were purchased from Sigma-Aldrich (St. Louis, MO, USA). Diphenyl-1-pyrenylphosphine (DPPP) was purchased from Cayman Chemical (Ann Arbor, MI, USA). Annexin V-FITC, 3-(4,5-dimethylthiazol-2-yl)-2,5-diphenyltetrazolium bromide (MTT), 2,3-bis­(2-methoxy-4-nitro-5-sulfophenyl)-2*h*-tetrazolium-5-carboxanilide (XTT), 4-amino-5-methylamino-2′,7′-difluorofluorescein diacetate (DAF-FM DA), and propidium iodide (PI) were purchased from Invitrogen (Grand Island, NY, USA). Roswell Park Memorial Institute (RPMI 1640), Dulbecco’s Modified Eagle Medium (DMEM) and fetal bovine serum (FBS) were purchased from Gibco (Grand Island, NY, USA). Uranyl acetate, sodium cacodylate, lead citrate, potassium ferrocyanide, glutaraldehyde, Polybed 812 resin, and osmium tetroxide were purchased from Electron Microscopy Sciences (EMS - Hatfield, PA, USA). CellTiter-Glo Luminescent Cell Viability reagent was purchased from Promega (Madison, WI, USA). The solvents and other reagents used were of analytical grade (P.A.) and used without purification.

### 4.2 Cell Culture

Promastigote forms of *L. (Leishmania) amazonensis* (MHOM/BR/75/Josefa) were maintained in Warren medium (Difco brain-heart infusion supplemented with hemin and folic acid −50 mg mL^–1^) pH 7.4, supplemented with 10% heat-inactivated FBS (56 °C, pH 7.4), and incubated at 25 °C in a Bio-Oxygen Demand (BOD) incubator. The experiments with promastigote forms were performed with 1 × 10^6^ parasites mL^–1^ in exponential growth phase (2 day culture). J774A.1 murine macrophages (Rio de Janeiro Cell Bank, Rio de Janeiro, Brazil) and L929 fibroblasts (clone NCTC 929, L cell, L-929; ATCC CCL1, Manassas, VA, USA) were cultured in supplemented RPMI 1640 medium (10% FBS, 5000 U mL^–1^ penicillin, and 5 mg mL^–1^ streptomycin) and supplemented DMEM (2 mM l-glutamine, 10% FBS, 5000 U mL^–1^ penicillin, and 5 mg mL^–1^ streptomycin), respectively, at 37 °C in a humidified atmosphere with 5% carbon dioxide (CO_2_). Intracellular amastigotes were obtained from J774A.1 macrophages infected with metacyclic promastigotes and cultured at 34 °C in a 5% CO_2_ atmosphere.

### 4.3 Synthesis of 4-[((4-Fluorophenyl)­amino)­methyl]-5-(4-nitrophenyl)-3-[(2*E*)-*N*′-(2-pyridinylmethylene)­hydrazinecarbonyl]­isoxazole (**4**)

The synthesis of 4-[((4-fluorophenyl)­amino)­methyl]-5-(4-nitrophenyl)-3-[(2E)-N′-(2-pyridinylmethylene)­hydrazinecarbonyl]­isoxazole (**4**) was performed according to the methodology described by Rosa et al.^25^ All the compounds were characterized based on ^1^H and ^13^C nuclear magnetic resonance (NMR) and high-resolution mass spectrometry (HRMS), with the purity of the compounds estimated by these techniques (>95%). The NMR spectra were presented at Supporting Information.

Stock solutions of **4** were prepared aseptically in DMSO and diluted in culture medium, so that the concentration of DMSO did not exceed 1% (v/v) in the assays.

#### 4.3.1 4-[((4-Fluorophenyl)­amino)­methyl]-5-(4-nitrophenyl)-3-[(2*E*)-*N*′-(2-pyridinylmethylene)­hydrazinecarbonyl]­isoxazole (**4**)

Orange solid; 82% yield; mp 240.2–243.3 °C; ^**1**^ **H NMR** (300.06 MHz, DMSO-*d*_6_) δ (ppm) 4.43 (*d*, 2H, NHCH_2_, *J* = 5.19 Hz), 5.95 (*t*, 1H, NHCH_2_, *J* = 5.21, 5.21 Hz), 6.57–6.61 (*m*, 2H, 4–F-C_6_H_4_), 6.89–6.95 (*m*, 2H,4-F C_6_H_5_), 7.43–7.47 (*m*, 1H, 2-C_5_H_4_N), 7.87–7.93 (*m*, 1H, 2-C_5_H_4_N), 7.96–7.99 (*m*, 1H, 2-C_5_H_4_N), 8.10 (*d*, 2H, 4-NO_2_–C_6_H_4_, *J* = 8.9 Hz), 8.42 (*d*, 2H, 4-NO_2_–C_6_H_4_, *J* = 8.6 Hz), 8.54 (*s*, 1H, NCH), 8.63 (*d*, 1H, 2-C_5_H_4_N, *J* = 4.7 Hz), 12.68 (*s*, 1H, NHN); ^**13**^ **C NMR** (75.45 MHz, DMSO-*d*_6_) δ (ppm) 36.1 (NHCH_2_), 113.6 (*d*, 4–F-C_6_H_4_, ^3^ *J*_C–F_ = 7.46 Hz), 115.2 (*d*, 4–F-C_6_H_4_, ^2^ *J*_C–F_ = 22.54 Hz), 120.1 (2-C_5_H_4_N), 124.4 (4-NO_2_–C_6_H_4_), 124.5 (2-C_5_H_4_N), 124.9 (C4), 128.7 (4-NO_2_–C_6_H_4_), 132.0 (4-NO_2_–C_6_H_4_), 137.0 (2-C_5_H_4_N), 144.9 (4–F-C_6_H_4_) 148.4 (4-NO_2_–C_6_H_4_), 149.6 (C=N), 150.0, 152.9 (2-C_5_H_4_N), 154.8 (*d*, 4–F-C_6_H_4_, *J* = 231.84 Hz), 155.7 (C3), 157.2 (C=O), 165.9 (C5); HRMS (ESI+): calcd for C_23_H_18_FN_6_O_4_^+^, [M + H]+: 461.1368, found 461.1374.

### 4.4 Antiproliferative Activity against Promastigotes of *L. amazonensis*

To determine the antiproliferative activity of isoxazole **4** on the growth of *L. amazonensis* promastigote forms, parasites were added to 96-well plates, in the absence or presence of different concentrations of compound **4** (1–100 μM), followed by incubation for 72 h at 25 °C. Cell viability assay was performed using the XTT reduction method.^69^ This method is based on the ability of mitochondrial dehydrogenase enzymes to convert the water-soluble tetrazolium salt into an orange-colored formazan derivative. Fifty microliters of XTT (0.5 mg mL^–1^) were added, and the cells were incubated for 4 h in the absence of light. Absorbance was measured in spectrophotometer (BIO-TEK Power Wave XS; Winooski, VT, USA) at 450 nm. The concentration that reduced 50% of parasite growth (IC_50_) was determined by nonlinear regression analysis.^70^

### 4.5 Antiproliferative Activity against Intracellular Amastigotes of *L. amazonensis*

To assess the activity of isoxazole **4** on intracellular amastigote forms, J774A.1 macrophages (5 × 10^5^ cells mL^–1^) were infected with metacyclic promastigotes (5 × 10^6^ parasites mL^–1^) and incubated for 24 h at 34 °C in a 5% CO_2_ atmosphere. Cells were washed with phosphate-buffered saline (PBS) to remove nonphagocytosed parasites, and infected macrophages were exposed to different concentrations of compound **4** (0.1–100 μM) and incubated for 48 h. Following treatment, cells were fixed with methanol for 20 min, stained with 10% Giemsa solution for 20 min and washed with distilled water. Visualization of 100 cells was performed using an optical microscope, and the infection index was established by multiplying the percentage of infected macrophages by the average number of parasites per macrophage. IC_50_ was determined by nonlinear regression analysis.^44^

### 4.6 *In Vitro* Cytotoxicity to Mammalian Cells

For cytotoxicity assessment, J774A.1 macrophages and L929 fibroblasts (5 × 10^5^ cells mL^–1^) were cultured in supplemented RPMI 1640 medium and DMEM, respectively, in 96-well plates and incubated for 24 h at 37 °C in a 5% CO_2_ atmosphere. Subsequently, cells were washed with PBS and exposed to increasing concentrations of isoxazole **4** (10–1000 μM) for 48 h. Fifty microliters of MTT (2 mg mL^–1^) were added, and the cells were incubated for 4 h in the absence of light. The resulting formazan crystals were solubilized in DMSO. Absorbance was measured in spectrophotometer (BIO-TEK Power Wave XS; Winooski, VT, USA) at 570 nm. The concentration that reduced 50% of cell viability (CC_50_) was determined by nonlinear regression analysis.^70,71^ The selectivity index was calculated as CC_50_/IC_50_.

### 4.7 Morphological and Ultrastructural Evaluation by Scanning Electron Microscopy (SEM) and Transmission Electron Microscopy (TEM)

For morphological and ultrastructural analyses, promastigote and intracellular amastigote forms were evaluated using incubation times corresponding to those applied in the respective antiproliferative assays for each parasite stage. For analysis of morphological changes by SEM, promastigotes were treated with concentrations corresponding to the IC_50_ and 2 × IC_50_ of compound **4** for 72 h at 25 °C. For visualization of intracellular amastigotes, J774A.1 macrophages (5 × 10^5^ cells mL^–1^) infected with promastigotes (5 × 10^6^ parasites mL^–1^) were treated with IC_50_ and 2 × IC_50_ of compound **4** for 48 h at 34 °C. Afterward, cells were washed with PBS and fixed by immersion in 2.5% glutaraldehyde in 0.1 M sodium cacodylate buffer for 24 h. Subsequently, the parasites were washed with 0.1 M sodium cacodylate buffer and, in the case of promastigotes, adhered to coverslips coated with poly-l-lysine for 1 h. Next, the parasites were washed with 0.1 M sodium cacodylate buffer and dehydrated in increasing concentrations of ethanol (30–100%). The samples were critical point dried with CO_2_ (Bal-Tec CPD 030; Bal-Tec AG, Balzers, Liechtenstein), coated with gold, and visualized on a high-resolution FEI Scios dual-beam electron microscope.

For analysis of ultrastructural changes by TEM, promastigotes and amastigotes were treated and fixed as described above. Next, the cells were postfixed with 1% osmium tetroxide, 0.8% potassium ferrocyanide, and 10 mM calcium chloride in 0.1 M sodium cacodylate buffer, dehydrated in increasing concentrations of acetone (30–100%), embedded in epoxy resin (Polybed 812), and polymerized at 60 °C for 72 h. Ultrathin sections of 60 nm were obtained using an ultramicrotome (PowerTomer; RMC, Tucson, AZ, USA), deposited on copper grids, contrasted with 5% uranyl acetate and 1.2% lead citrate, and finally visualized on a JEOL JEM 1400 transmission electron microscope.

### 4.8 Isolation of Intracellular Amastigote Forms

J774A.1 macrophages were infected with promastigotes (10 parasites per host cell) and incubated for 48 h at 34 °C in a 5% CO_2_ atmosphere. Subsequently, the infected macrophages were treated with IC_50_ and 2 × IC_50_ of compound **4** for 24 h. After treatment, infected macrophages were removed using a cell scraper and lysed by extrusion with a syringe and a 30-gauge needle.^72^ Amastigotes were separated from unlysed macrophages and debris by differential centrifugation (1000 rpm for 1 min; 5000 rpm for 5 min), the supernatant was collected and washed with PBS, and the amastigotes were subjected to different assays.

### 4.9 Assessment of Mechanism of Action of Isoxazole **4** in *L. amazonensis* Promastigotes and Intracellular Amastigotes

To elucidate the mechanism of action of the isoxazole **4** in *L. amazonensis*, promastigotes and intracellular amastigotes were treated with IC_50_ and 2 × IC_50_ of the compound and incubated at 25 and 34 °C, respectively, for 24 h. For fluorometric assays, parasites were distributed in black 96-well plates, and the fluorescence measurements were performed at a multilabel microplate reader (Victor X3; PerkinElmer, Waltham, MA, USA). The number of parasites was counted in a Neubauer hemocytometer and adjusted to 1 × 10^6^ parasites mL^–1^.

For flow cytometry assays, data acquisition and analysis were performed on a FACSCalibur flow cytometer (Becton-Dickinson, Rutherford, NJ, USA) equipped with CellQuest software (Joseph Trotter, Scripps Research Institute, La Jolla, CA, USA). A total of 30,000 events were acquired in the region previously established with the parasite.

#### 4.9.1 Detection of Total Reactive Oxygen Species (ROS)

To assess the effect of compound **4** in ROS content, treated promastigotes and intracellular amastigotes were incubated in the dark with H_2_DCFDA (10 μM) for 45 min. H_2_DCFDA is cell-permeable, and upon cleavage by intracellular esterases and oxidation by ROS, the nonfluorescent probe H_2_DCFDA is converted to the highly fluorescent product 2′,7′-dichlorofluorescein (DCF). Fluorescence was measured at λ_ex_ = 488 nm and λ_em_ = 530 nm.^73^ Additionally, the increase in ROS was observed under an Olympus BX51 fluorescence microscope (Olympus), and images were captured with a UC30 camera (Olympus). H_2_O_2_ (50 μM) was used as positive control.

#### 4.9.2 Detection of Nitric Oxide (NO)

To investigate the NO content, treated promastigotes and intracellular amastigotes were incubated in the dark with DAF-FM DA (1 μM) for 30 min. DAF-FM DA is cell-permeable and is hydrolyzed to DAF-FM by intracellular esterases, reacts with NO, and forms a triazole compound that emits fluorescence.^74^ Then, the parasites were washed and resuspended in PBS, and incubated for 15 min. Fluorescence was measured at λ_ex_ = 495 nm and λ_em_ = 515 nm.^57^ H_2_O_2_ (50 μM) was used as positive control.

#### 4.9.3 Lipid Peroxidation Assessment

To analyze lipid peroxidation, parasites were incubated in the dark with DPPP (50 μM) for 15 min. DPPP is a nonfluorescent molecule that reacts with lipid hydroperoxides forming a fluorescent product DPPP-O.^75,76^ Fluorescence was measured at λ_ex_ = 380 nm and λ_em_ = 460 nm.^77^ H_2_O_2_ (50 μM) was used as positive control.

#### 4.9.4 Cytoplasmic Lipid Droplets Detection

To determine lipid droplet accumulation, parasites were incubated in the dark with Nile red (10 μg mL^–1^), a lipophilic marker that associates with neutral lipid molecules, for 30 min. Fluorescence was measured at λ_ex_ = 485 nm and λ_em_ = 535 nm.^57^ H_2_O_2_ (50 μM) was used as positive control.

#### 4.9.5 Mitochondrial Membrane Potential (ΔΨm) Assessment

To determine the ΔΨm, parasites were incubated in the dark with the fluorescent probe that accumulates in the mitochondria, Rh123 (5 μg mL^–1^), for 15 min at 37 °C. Parasites were washed twice, resuspended in 0.9% saline solution and incubated for 30 min at 37 °C. The assessment was performed using flow cytometry. The mitochondrial oxidative phosphorylation uncoupler CCCP (100 μM) was used as positive control.^77^

#### 4.9.6 Intracellular Adenosine Triphosphate Levels (ATP) Measurement

ATP levels were determined using Cell Titer-Glo Luminescent Cell Viability Assay, according to the manufacturer instructions. Treated parasites were washed, resuspended in PBS, and incubated in the dark for 10 min in white 96-well plates with an equal volume of the CellTiter-Glo reagent.^72^ Luminescence was measured using a microplate reader (SpectraMax L; Molecular Devices, USA). KCN (200 μM) was used as positive control.

#### 4.9.7 Cell Size Assessment

To analyze cell size, parasites were washed and resuspended in PBS and quantified by flow cytometry. Forward scatter height (FSC-H) was used as an indicator of cell size. Miltefosine (40 μM) was used as positive control.^78^

#### 4.9.8 Phosphatidylserine Exposure Detection

To detect phosphatidylserine exposure, parasites were incubated in the dark with 5 μL of Annexin V-FITC in 100 μL of binding buffer (140 mM NaCl, 5 mM CaCl_2_, 10 mM HEPES-Na, pH 7.4) for 15 min at room temperature. Subsequently, 400 μL of binding buffer and PI (2 μg mL^–1^) were added. The assessment was performed using flow cytometry. Cells labeled with Annexin V (positive or negative PI) were considered apoptotic, and cells positive only for PI were considered necrotic. Miltefosine (40 μM) was used as positive control.^73,79^

#### 4.9.9 Cell Membrane Integrity Assessment

To analyze the integrity of cell membrane, parasites were incubated in the dark with PI (2 μg mL^–1^) for 5 min. Membrane cell rupture allows the entry of PI and its binding with nucleic acids. The assessment was performed using flow cytometry. Digitonin (40 μM) was used as positive control.^28^

#### 4.9.10 Autophagic Vacuoles Detection

To quantify autophagic vacuoles, parasites were incubated in the dark with MDC (0.05 mM) for 1 h at 37 °C. MDC accumulates in autophagic vacuoles by trapping ions and interacting with the lipids of their membrane.^80^ All treatments were also evaluated with wortmannin (1 μM), a potent inhibitor of phosphatidylinositol 3-kinase (PI3–K), a family of enzymes that regulate autophagy.^51,81^ After incubation, parasites were washed with PBS, and fluorescence was measured at λ_ex_ = 380 nm and λ_em_ = 525 nm.^57^ Additionally, the accumulation of autophagic vacuoles was observed under an Olympus BX51 fluorescence microscope (Olympus), and images were captured with a UC30 camera (Olympus). For positive control, parasites were subjected to stress due to nutritional scarcity by diluting the culture medium in PBS.^82^

#### 4.9.11 Acidic Compartments Detection

To assess acidic compartments, parasites were incubated with acridine orange (1 μg mL^–1^) for 15 min at room temperature. After incubation, parasites were washed and resuspended in PBS. Acridine orange in its monomer form emits green fluorescence, but when accumulated in acidic vesicles - low pH environments - acridine orange molecules are protonated, and the electrical charge hinders the exit of the marker to the cytoplasm, forming dimers emitting red fluorescence.^83^ Assessment was performed using flow cytometry, and the ratio between red and green fluorescence was calculated and related to the acidic compartments content present in the cells.^84^ H_2_O_2_ (50 μM) was used as positive control.

### 4.10 *In Silico* Studies

The SwissADME web tool^30^ was employed to calculate the physicochemical properties of isoxazole **4**, considering the Lipinski’s rule of five, which relates pharmacokinetic and physicochemical parameters,^31^ and Veber’s rule,^32^ in addition to the presence of pan-assay interference compounds (PAINS).^85^ The molecule’s solubility was predicted by Log S.^86^ The software also allowed obtaining Bioavailability Radar and *Brain Or IntestinaL EstimateD permeation* method (BOILED-Egg) graphs.

### 4.11 Statistical Analysis

Data were expressed as mean ± standard deviation (s.d.) of at least three independent experiments. Data were analyzed using GraphPad Prism 8.0.1 software (GraphPad, San Diego, CA, USA). Statistical analysis was performed using one- or two-way analysis of variance (ANOVA) followed by Tukey’s multiple comparisons test. Values of *p* < 0.05 were considered statistically significant.

## Supplementary Material



## Notes

1H and ^13^C NMR spectra of the isoxazole derivative (compound **4**); SwissADME analysis of compound **4**, including Bioavailability Radar and BOILED-Egg plots (PDF)
