# Supplementary material for: Isoxazole Derivative Induces Apoptosis-like Death and Autophagy through Oxidative Stress in Leishmania amazonensis
Source: ACS Omega. 2026 May 2;11(18):26310–27. doi: 10.1021/acsomega.5c11341 (PMC13177027; doi:10.1021/acsomega.5c11341)
Supplement: Supplementary file 1 [file ao5c11341_si_001.pdf]

Supporting Information

Isoxazole derivative induces apoptosis-like death and autophagy through oxidative stress in *Leishmania amazonensis*

Amanda Beatriz Kawano Bakoshi[a], Rayanne Regina Beltrame Machado[a], Karlos Eduardo Pianoski[b], Samara Mendes de Souza Melo[b], Fernanda Andreia Rosa[b], Sueli de Oliveira Silva Lautenschlager[a], Tania Ueda-Nakamura[a], Celso Vataru Nakamura[a], Danielle Lazarin-Bidóia[a,*].

[a] Laboratory of Technological Innovation in the Development of Drugs and Cosmetics, State University of Maringá, 87020-900, Maringá, PR, Brazil.

[b] Department of Chemistry, State University of Maringá, 87020-900, Maringá, PR, Brazil.

*Corresponding Author:

Danielle Lazarin-Bidóia

Laboratory of Technological Innovation in the Development of Drugs and Cosmetics

State University of Maringá

Avenida Colombo, 5790, 87020-900, Maringá, Paraná, Brazil

Phone: +55 (44) 3011-5012

E-mail: dlbidoia@gmail.com

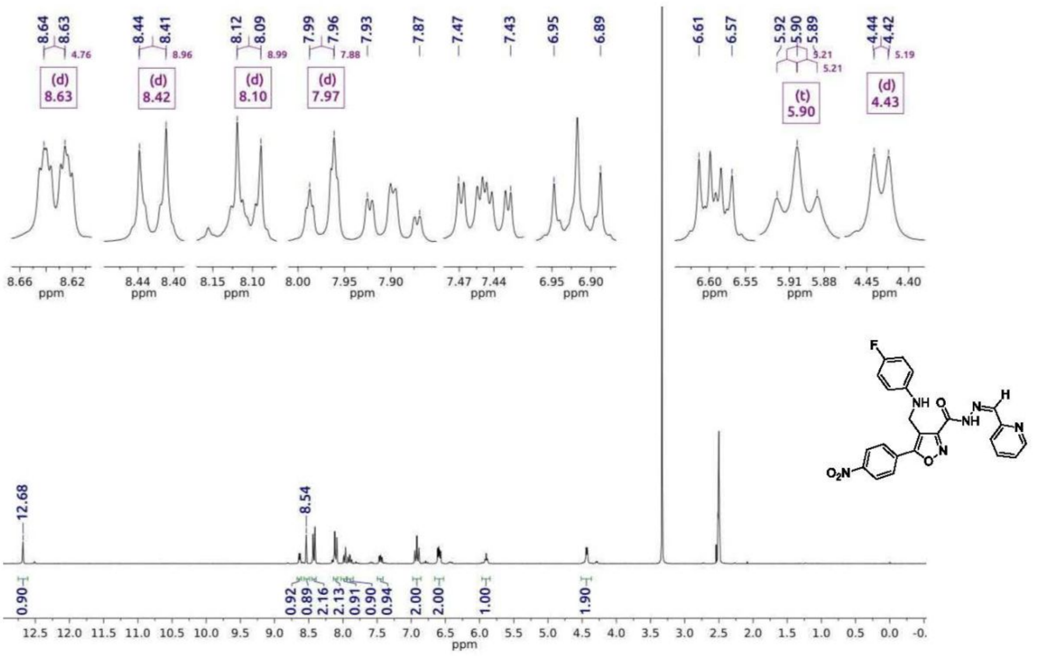

Figure S1. ^{1}H NMR spectrum of isoxazole **4** (DMSO-d6, 300 MHz).

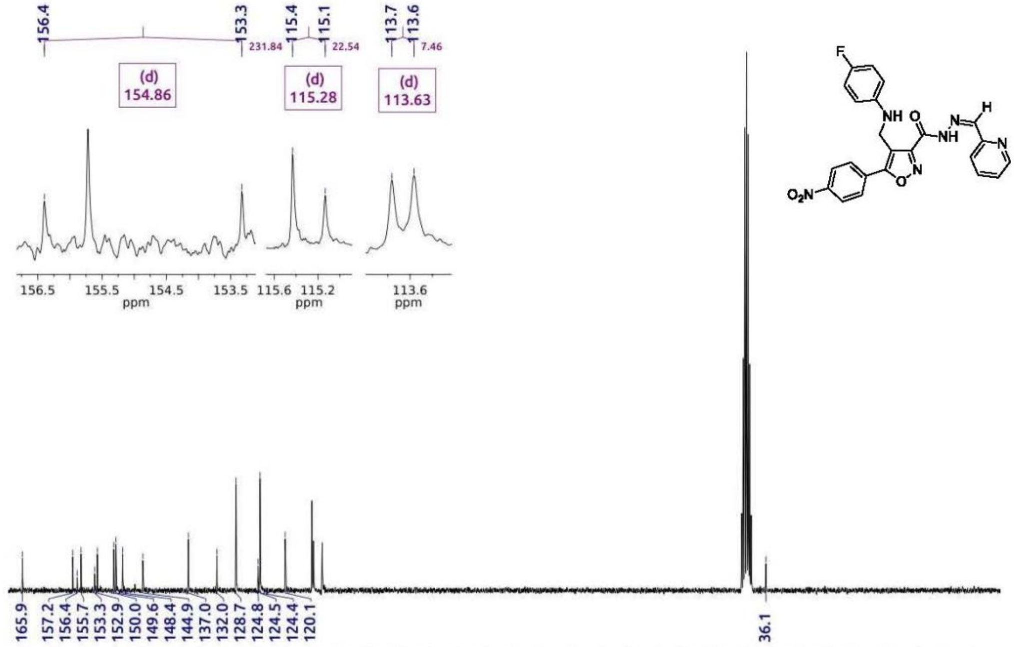

Figure S2. ^{13}C NMR spectrum of isoxazole **4** (DMSO-d6, 75 MHz).

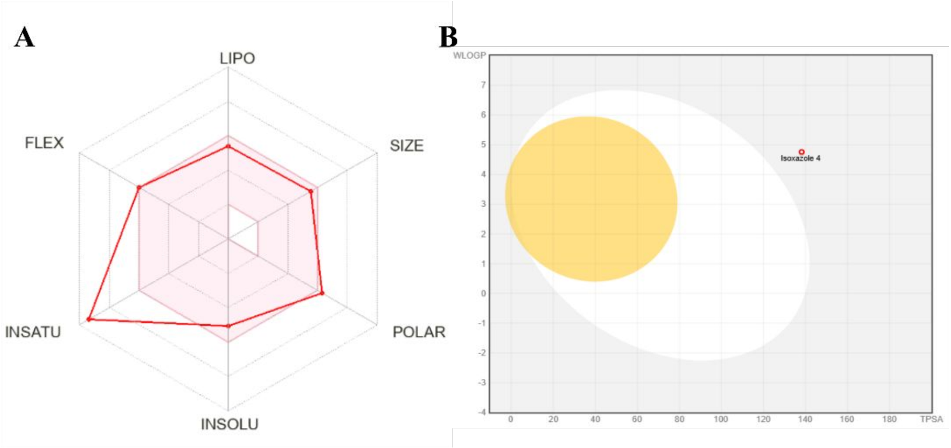

Figure S3. (A) Bioavailability Radar plot for compound isoxazole **4** predicted using the SwissADME tool, indicating the properties related to drug-likeness. The pink area represents the optimal range for each property: lipophilicity (LIPO), molecular size (SIZE), polarity (POLAR), solubility (INSOLU), flexibility (FLEX), and saturation (INSATU). (B) BOILED-EGG plot showing the probability of human intestinal absorption and blood-brain barrier permeation. The red dot indicates that the compound isoxazole **4** is predicted not to be a substrate for P-glycoprotein efflux. WLOGP: lipophilicity parameter; TPSA: topological polar surface area.
